# Development of brain mechanisms for processing affective touch

**DOI:** 10.3389/fnbeh.2014.00024

**Published:** 2014-02-04

**Authors:** Malin Björnsdotter, Ilanit Gordon, Kevin A. Pelphrey, Håkan Olausson, Martha D. Kaiser

**Affiliations:** ^1^Department of Physiology, Institute for Neuroscience and Physiology, University of GothenburgGothenburg, Sweden; ^2^Child Study Center, Yale UniversityNew Haven, CT, USA

**Keywords:** fMRI, touch, brain, children, development

## Abstract

Affective tactile stimulation plays a key role in the maturation of neural circuits, but the development of brain mechanisms processing touch is poorly understood. We therefore used functional magnetic resonance imaging (fMRI) to study brain responses to soft brush stroking of both glabrous (palm) and hairy (forearm) skin in healthy children (5–13 years), adolescents (14–17 years), and adults (25–35 years). Adult-defined regions-of-interests in the primary somatosensory cortex (SI), secondary somatosensory cortex (SII), insular cortex and right posterior superior temporal sulcus (pSTS) were significantly and similarly activated in all age groups. Whole-brain analyses revealed that responses in the ipsilateral SII were positively correlated with age in both genders, and that responses in bilateral regions near the pSTS correlated significantly and strongly with age in females but not in males. These results suggest that brain mechanisms associated with both sensory-discriminative and affective-motivational aspects of touch are largely established in school-aged children, and that there is a general continuing maturation of SII and a female-specific increase in pSTS sensitivity with age. Our work establishes a groundwork for future comparative studies of tactile processing in developmental disorders characterized by disrupted social perception such as autism.

## Introduction

Touch is a multifaceted stimulus, activating a range of mechanoreceptors and neural pathways depending on site and mode of stimulation (Abraira and Ginty, 2013). Tactile information not only conveys characterization of external stimuli (the *sensory-discriminative* dimension), such as in object manipulation, but touch can also be pleasant and social (the *affective-motivational* dimension) (Keysers et al., 2010; Morrison et al., 2010). A growing body of animal studies shows that postnatal experiences actively shape central sensory circuits in a complex interplay between afferent input (Koch et al., 2012), and has established that parental affective tactile behavior during early stages of neural development, such as licking and grooming, may have a profound impact on behavior in the adult animal (Hofer, 1995; Zhang and Meaney, 2010; Bagot et al., 2012; Suderman et al., 2012). In primates, touch is considered to play a crucial role during development (Harlow, 1958; Corbetta and Snapp-Childs, 2009; Cascio, 2010; Feldman et al., 2010) and disrupted processing of touch has been linked to psychiatric illness and neurodevelopmental disorders (Cascio, 2010; Voos et al., 2013). Despite the potential influence of touch during development, however, very little is known about the development of brain mechanisms for processing touch.

In healthy adults, innocuous, non-painful touch activates cutaneous low-threshold mechanoreceptors (LTMRs) (Vallbo et al., 1993, 1999; Olausson et al., 2002; Mountcastle, 2005; Abraira and Ginty, 2013). The resulting signals may travel through one of two kinds of afferent fibers to the spinal cord: thick myelinated Aβ afferents or thin unmyelinated C tactile (CT) fibers (Björnsdotter et al., 2010; Abraira and Ginty, 2013). The LTMRs associated with Aβ afferents innervate the entire body (Goodwin and Wheat, 2008) and are key in coding the sensory-discriminative dimension of touch. CT afferents have been identified exclusively in the hairy skin and appear to be absent in glabrous skin (i.e., the palms or the soles of the feet) (Vallbo et al., 1999; Liu et al., 2007). The specific function of CT afferents is largely unknown, but the fibers respond vigorously to pleasant types of tactile experiences, such as slow (1–10 cm/s), gentle stroking of the skin (Vallbo et al., 1993; Löken et al., 2009) and the system is associated with the affective-motivational dimension of touch (Morrison, 2012).

Peripheral and central processing of Aβ-mediated touch is exceptionally well-studied in animals and adult humans. Many decades of research has established that the contralateral primary (SI) and bilateral secondary (SII) somatosensory cortices are key regions in basic touch processing (Qi et al., 2008). Nonetheless, research on the development of somatosensory function in humans is surprisingly scant. The handful of studies that examined tactile processing in children suggests that the most basic mechanisms of somatosensory processing may be present at a very young age. A study of preterm infants showed that electroencephalography (EEG) responses to somatosensory stimuli are unspecific until 35–37 weeks of gestation, when the capability of neural circuits to distinguish painful from non-painful stimuli emerge (Fabrizi et al., 2011). Sedated infants aged 3–96 months are reported to activate the postcentral gyrus, likely corresponding to SI, in response to rubbing of the hand (Souweidane et al., 1999). In older children, aged 11–17 years, tactile stimulation of the hand activated SI (Van de Winckel et al., 2013). These studies demonstrate that fundamental brain mechanisms are in place, but also raise the question of the degree to which touch processing in somatosensory brain regions is adult-like already in children.

Studies in adult neuronopathy patients who lack Aβ fibers have shown that pure CT stimulation activates the insular cortex but not the somatosensory regions (Olausson et al., 2002, 2008; Björnsdotter et al., 2009). CT-targeted stimulation also activates key nodes of the “social brain” in adults, including the posterior superior temporal sulcus (pSTS) and prefrontal regions (Voos et al., 2013; Gordon et al., 2013). Activations in the right and left superior temporal gyrus, near the STS, were found in children aged 11–17 years in response to gentle stroking of the dorsal part the hand with a sponge cotton cloth (Van de Winckel et al., 2013). However, it is not clear whether the STS is recruited in younger children. Another recent study examined brain responses to gentle tactile stimuli of the glabrous palm of the hand in infants of different ages (Kida and Shinohara, 2013a). This study found that the prefrontal cortex was activated more in response to stimuli by soft velvet than to a wooden stimulus in 10-month olds but not in younger infants. This finding suggests that specificity of prefrontal circuits involved in affective processing may emerge during infancy, and raises the question of when these circuits reach an adult-like stage. Moreover, the study examined responses to stimulation of the palm of the hand, lacking CT afferents, in effect comparing brain responses to two different types of Aβ LTMR. Here, we were specifically interested in characterizing developmental effects of brain responses to CT-targeted touch. Moreover, insular cortex responses to CT-targeted stimuli have not been previously examined in children.

Taken together, previous research suggests that some basic brain mechanisms processing sensory-discriminative as well as affective-motivational touch are in place at a young age. It is critical, however, to further characterize the brain mechanisms of touch processing in the developing brain in order to fully understand the link between tactile experiences and behavior. In particular, a detailed understanding of the normative developmental trajectories is a necessary step in the understanding of deviating processing in clinical populations and the putative link between development and disorders associated with disrupted social processing such as autism (Voos et al., 2013).

## Methods

### Participants

Twenty two healthy adults (nine females, mean age = 24.52 years, range 19–35 years), 10 healthy children (six females, mean age = 10.68, range 5.6–13.3 years) and 9 healthy adolescents (four females, mean age = 14.95, range 13.5–17 years) were studied. Each participant or their parent or guardian provided written consent according to a protocol approved by the Yale School of Medicine Human Investigations Committee.

### Stimuli

The tactile stimuli consisted of manual strokes with a 7-cm wide watercolor brush applied with slow strokes at a CT optimal velocity (8 cm/s) (Löken et al., 2009). The stimuli were applied to the hairy skin of the forearm (Arm; *CT-targeted touch*) and to the palm of the hand (Palm; *Aβ targeted touch*). In each participant, 8 cm of the arm and 4 cm of the palm were marked to control for the length of stimulated skin, and two trained experimenters administered the stimuli.

### Paradigm

**Figure 1:**
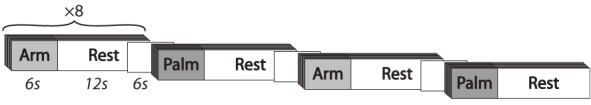
**Experimental paradigm**.

Continuous brushing (back and forth) was applied to the right palm or forearm according to a block design (Figure 1). Each block included 6 s of touching followed by 12 s of rest. Six seconds of Baseline rest followed each block. Blocks containing each condition (Arm, Palm) were repeated eight times. The participants were instructed to lie still with eyes closed during the procedure, and to focus on the tactile sensation.

### Imaging protocol

fMRI brain scans were acquired on a Siemens 3T Tim Trio scanner (at the Yale University Magnetic Resonance Research Center). Anatomical images were collected using a T1-weighted MPRAGE sequence (*TR* = 1230 ms; *TE* = 1.73 ms; FOV = 256 mm; image matrix 2562; voxel size = 1 × 1 × 1 mm). Whole-brain functional images were obtained using a single-shot, gradient-recalled echo planar pulse sequence (*TR* = 2000 ms; *TE* = 25 ms; flip angle = 60°; FOV = 220 mm; image matrix = 642; voxel size = 3.4 × 3.4 × 4.0 mm; 34 slices).

### fMRI data processing and analysis

Data were processed in BrainVoyager QX 2.0.08 (Brain Innovation, Maastricht, The Netherlands). Functional data preprocessing included slice time correction (using sinc interpolation), three-dimensional rigid-body motion correction using trilinear-sinc interpolation, spatial smoothing with a FWHM 4-mm Gaussian kernel, linear trend removal, and temporal high-pass filtering (GLM with Fourier basis set, using two cycles per time course). Functional images were co-registered to within-session anatomical images and normalized to Talairach space. In each participant, estimated motion plots and cine loops were inspected for head motion greater than 2 mm of translation in any direction or two degrees of rotation about any axis. Also, no participant had rotation or translation exceeding 1 mm between two consecutive volumes or 2 mm integrated over four consecutive volumes. A general linear model (GLM) analysis was performed in each participant. Regressors were defined as boxcar functions convolved with a double-gamma hemodynamic response function (HRF). Six motion predictors were included as predictors of no-interest.

#### Whole-brain activations

Whole-brain random-effects group-level GLM analyses were conducted in each group (children, adolescents, adults) for the contrasts Arm touch > Rest and Palm touch > Rest. All group-level analyses were restricted to voxels within the Montreal Neurological Institute (MNI) template brain normalized to Talairach space. In children and adolescents, the results were assessed at *p* < 0.01 and corrected for multiple comparisons with a cluster level threshold estimated through the Brain Voyager QX cluster-level statistical threshold estimator plug-in (Forman et al., 1995; Goebel et al., 2006). Using 1000 iterations of a Monte Carlo simulation, the relative frequency of each cluster size (*k*) was assessed. A cluster-corrected threshold was set at α < 0.05 for each contrast. Given the higher power of the larger group, the adult maps were thresholded at a higher threshold of *p* < 0.001 before cluster level correction. The cluster-level threshold was not applied in the displayed images for visualization purposes.

#### Region-of-interest analysis

To examine the extent to which brain responses in children and adolescents were adult-like, we performed a region-of-interest (ROI) analysis. Here, we first examined the network of sensory-discriminative brain regions, associated with stimulation of Aβ afferents, with focus on contralateral (left) SI and bilateral SII (Donkelaar and Brabec, 2011). Since all types of touch activate Aβ afferents, and hence the somatosensory cortices, we maximized power by examining the main effect of touch regardless of condition (contrast Arm + Palm > Rest). We performed a whole-brain random effects GLM analysis for main effect of touch in adults (*p* < 0.001, cluster level correction for multiple comparisons), and defined all significant clusters located to the left SI and bilateral SII as ROIs. We then examined brain regions associated with the CT-targeted, affective-motivational dimension of touch, including the contralateral (left) insular cortex (Olausson et al., 2002; Björnsdotter et al., 2009), the right pSTS and the prefrontal cortex (Bennett et al., 2013; Gordon et al., 2013; Kida and Shinohara, 2013b). Here, we examined Arm stroking (CT-targeted stimuli) in isolation. ROIs were extracted from the adult contrast Arm > Rest (*p* < 0.001, cluster level correction for multiple comparisons), and significant voxel clusters located in the left insula, the right pSTS and the prefrontal cortex were identified. In each of the ROIs defined in the adult group, we extracted individual voxel-average brain responses (β values) in all groups. We then performed a *post-hoc* Three-Way (children, adolescents, adults) analysis of variance (ANOVA) to examine differences between the groups, and a correlation analysis to assess correlations between β values and age. We also tested for gender differences in each ROI. Since the sex ratios were unbalanced in children (six females, three males) and adolescents (four females, six males), we combined these participants into one group (children/adolescents) in this analysis.

#### Whole-brain correlation analysis

In order to identify age-related changes in brain processing of touch, we conducted a whole-brain, voxel-wise correlation analysis assessing linear correlations between individual brain responses and age. Here, we also examined gender-specific developmental effects by repeating the analysis in females and males, separately. In order to match males and females in terms of power and age, we excluded the youngest male (5 years old) and two randomly selected adults. This resulted in 19 subjects of each gender, with no significant differences in age (*p* = 0.586, two-sample *t*-test).

## Results

### Whole-brain activations

**Figure 2:**
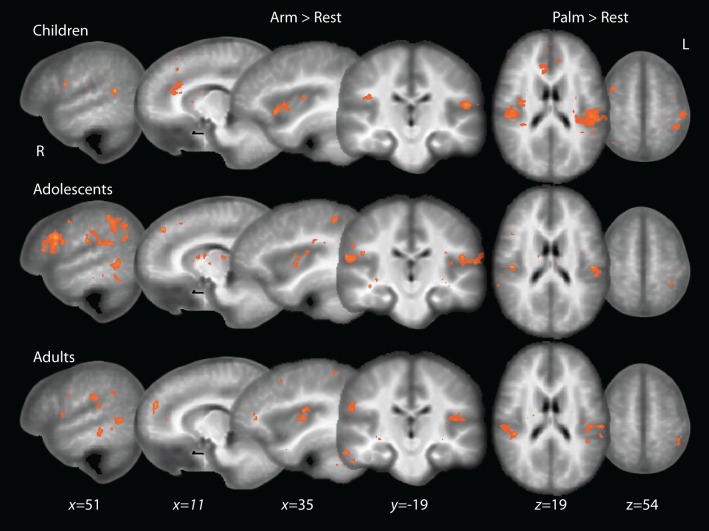
**Whole-brain activations to touch in children, adolescents and adults**. Adolescent and child maps are thresholded at *p* < 0.01, uncorrected for multiple comparisons. Adult maps are shown at a thresholded of *p* < 0.001, uncorrected.

The adult group activated all expected regions in response to touch (Arm touch: *p* < 0.001, *k* = 11; Palm touch: *p* < 0.001, *k* = 10), including the left (contralateral) SI, bilateral SII, left insular cortex, several prefrontal cortex areas and a cluster of voxels extending into the pSTS (with the peak in the middle temporal gyrus, MTG) (Figure 2, Table 1). In children and adolescents, only the left (contralateral) SII (Palm as well as Arm touch) and insular cortex (Arm touch) consistently passed the whole-brain threshold (children Arm touch: *p* < 0.01, *k* = 18; Palm touch: *p* < 0.01, *k* = 18; adolescents Arm touch: *p* < 0.01, *k* = 22; Palm touch: *p* < 0.01, *k* = 19; Table 1). However, the lack of significant responses was likely related to reduced power compared to adults: voxels passing the significance threshold but not the cluster-correction threshold were found in both children and adolescents in pSTS/MTG (for Arm brush) and SI (for Palm brush) (Figure 2).

**Table 1:** **Brain regions showing significant activations in response to touch**.

**Arm > Rest**	**Region**	***x,y,z***	***T***	***p***	**Nr Voxels**
Children	L anterior cingulate	−7, 34, 9	5.50	<0.001	34
	L insular cortex	−34, 16, −3	5.05	<0.001	28
	L SII	−58, −26, 18	7.76	<0.001	59
Adolescents	R SII	41, −26, 24	12.86	<0.001	267
	R inferior frontal gyrus	57, 10, 18	7.44	<0.001	184
	R pSTS/MTG	38, −44, 0	7.25	<0.001	33
	R precentral gyrus	41, −2, 42	7.57	<0.001	67
	R angular gyrus	41, −62, 36	5.50	<0.001	23
	R caudate	11, 7, 15	7.73	<0.001	37
	L caudate	−13, −2, 15	10.21	<0.001	62
	L SI	−34, −44, 45	5.07	<0.001	25
	L SII, insula	−61, −32, 21	7.00	<0.001	134
	L inferior parietal Lobule	−46, −44, 42	6.93	<0.001	28
	L precentral gyrus	−58, 1, 21	7.12	<0.001	60
Adults	R pSTS/MTG	56, −50, 3	5.12	<0.001	34
	R SII	41, −29, 21	5.98	<0.001	50
	R middle temporal gyrus	56, −26, −9	5.29	<0.001	13
	R inferior frontal gyrus	29, 31, 9	5.99	<0.001	97
	R cerebellum	26, −59, −27	5.94	<0.001	20
	R superior frontal gyrus	11, 49, 27	6.05	<0.001	19
	L medial frontal gyrus	−7, 28, 30	5.17	<0.001	17
	L superior frontal gyrus	−10, 22, 51	6.11	<0.001	16
	L cerebellum	−19, −68, −27	4.73	<0.001	12
	L SI	−28, −44, 54	4.82	<0.001	14
	L SII	−46, −32, 12	5.94	<0.001	20
	L insula	−37, −8, 12	6.13	<0.001	39
	L SII	−49, −23, 24	6.94	<0.001	43
**Palm > Rest**	**Region**	***x,y,z***	***T***	***P***	**Nr Voxels**
Children	R SII	47, −26, 18	6.55	<0.001	55
	R insula	38, −2, 0	7.37	<0.001	38
	R putamen	26, 4, 9	5.97	<0.001	27
	R anterior cingulate	11, 25, 27	5.51	<0.001	23
	L SII, insula	−46, −26, 18	10.44	<0.001	162
	L SI	−37, −41, 57	7.89	<0.001	131
Adolescents	R supramarginal gyrus	56, −47, 33	6.28	<0.001	30
	R cerebellum	26, −56, −39	5.68	<0.001	25
	R thalamus	2, −2, 9	8.09	<0.001	33
	R posterior cingulate	8, −53, 24	7.63	<0.001	29
	L SII	−46, −20, 18	6.28	<0.001	51
Adults	R inferior parietal Lobule	50, −41, 27	5.53	<0.001	16
	R SII	41, −29, 21	5.53	<0.001	25
	R cerebellum	26, −59, −27	6.30	<0.001	102
	L cerebellum	−22, −56, −33	7.08	<0.001	73
	L thalamus	−19, −11, 15	5.69	<0.001	11
	L SI	−43, −38, 45	4.88	<0.001	18
	L insula	−37, −14, 6	7.06	<0.001	26
	L SII	−62, −26, 24	5.99	<0.001	56
	L postcentral gyrus	−46, −32, 39	5.55	<0.001	19
	L inferior parietal lobule	−52, −44, 36	5.70	<0.001	10

Talairach coordinates and statistics refer to the voxel with the maximum signal change in each region. Abbreviations: right (R), left (L), primary somatosensory cortex (SI), secondary somatosensory cortex (SII), superior temporal sulcus (STS), medial temporal gyrus (MTG).

### Region-of-interest analysis

**Figure 3:**
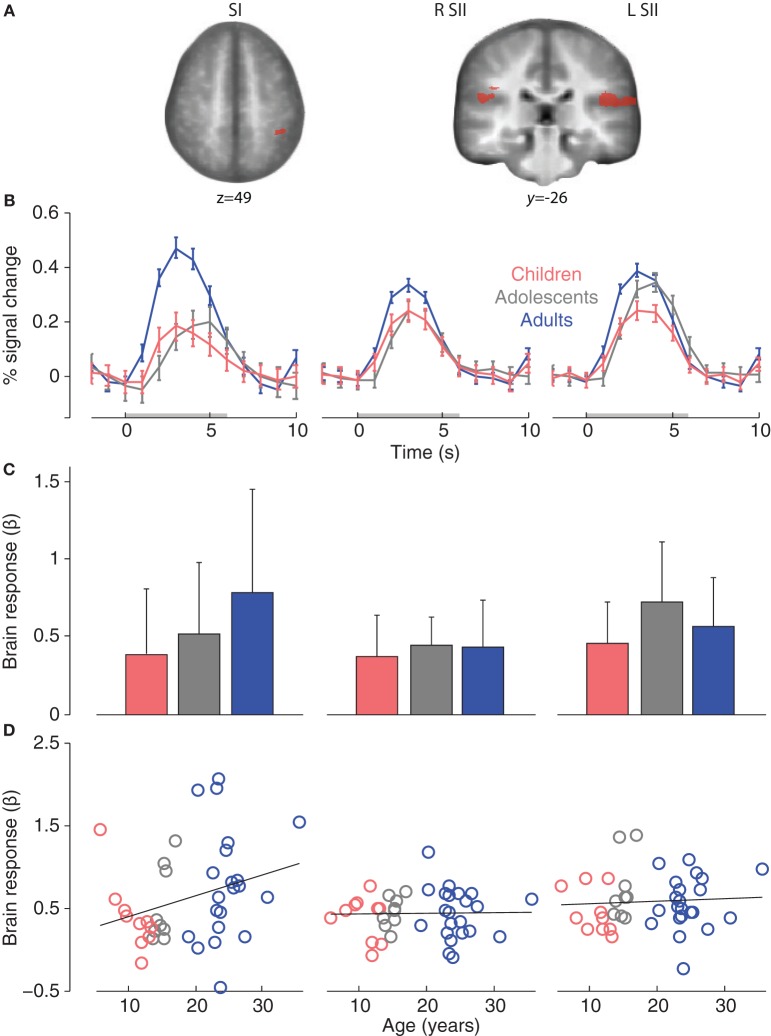
**(A)** Adult somatosensory ROIs for main effect of touch (Arm touch + Palm touch > Rest, *p* < 0.001, *k* = 12). **(B)** Group mean ROI event-related brain responses (to Arm touch + Palm touch). **(C)** Group mean ROI-average brain responses. The error bars show standard deviations. **(D)** Correlations between age and individual brain ROI-average response. Abbreviations: primary somatosensory cortex (SI), secondary somatosensory cortex (SII).

We examined activations in adult ROIs in order to assess the degree to which brain responses in children and adolescent were adult-like. First, sensory-discriminative ROIs were identified in the adult group for main effect of touch (Arm touch and Palm touch > Rest; *p* < 0.001, *k* = 12). Highly significant clusters were found with peak coordinates in the SII (bilateral operculum) and left SI (Table 2; Figure 3A).

**Table 2:** **Adult somatosensory ROIs and activations for main effect of touch (Arm touch + Palm touch > Rest) in all age groups**.

**Adult ROI**	***x,y,z***	**Nr Voxels**	**Adults**		**Adolescents**		**Children**	
			***T***	***P***	***T***	***p***	***T***	***p***
L SI	−37, −41, 45	17	5.05	<0.001	3.39	0.010	2.82	0.020
R SII	41, −29, 21	44	6.55	<0.001	7.74	<0.001	4.42	0.002
L SII	−49, −23, 24	93	6.96	<0.001	5.66	<0.001	5.39	<0.001

Talairach x,y,z coordinates and statistics refer to the voxel with the maximum signal change in each region. Abbreviations: region of interest (ROI), right (R), left (L), secondary somatosensory cortex (SII), primary somatosensory cortex (SII).

All adult somatosensory ROIs were significantly activated in children and adolescents (*p* < 0.05, small-volume correction in respective ROI; Table 2). Event-related brain responses were largely similar in amplitude and timing across age groups (Figure 3B), with the exception of SI in which responses were reduced in amplitude in children and adolescents compared to adults. Nevertheless, a *post-hoc* ANOVA showed that there was no significant effect of age group on the brain responses in SI (*F* = 1.9, *p* = 0.17) or any of the other adult ROIs (R SII *F* = 0.21, *p* = 0.809; L SII *F* = 1.7, *p* = 0.202; Figure 3C). There were no significant correlations between age and brain responses in any of the somatosensory ROIs (R SII *r* = 0.02, *p* = 0.909; L SII *r* = 0.07, *p* = 0.673; Figure 3D) although there was a non-significant trend toward a positive correlation between age and brain responses in the adult SI (*r* = 0.28, *p* = 0.072). There were no significant differences between females and males in adults or children/adolescents (all *ps* > 0.1).

**Figure 4:**
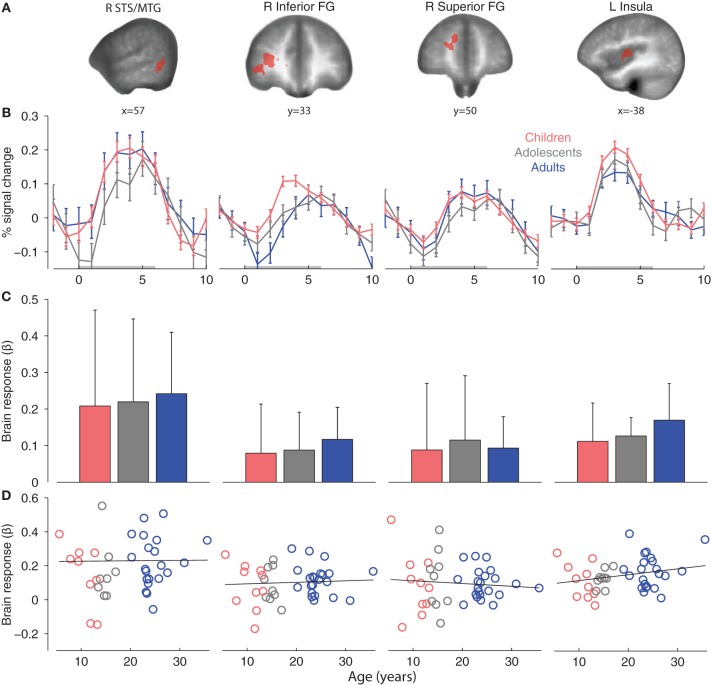
**(A)** Adult affective-motivational ROIs (Arm touch > Rest, *p* < 0.001, *k* = 11). **(B)** Group mean ROI event-related brain responses to Arm touch. **(C)** Group mean ROI-average brain responses. The error bars show standard deviations. **(D)** Correlations between age and individual brain ROI-average response. Abbreviations: middle temporal gyrus (MTG), superior temporal sulcus (STS), frontal gyrus (FG).

Second, we defined affective-motivational touch ROIs in the adult group for the contrast Arm > Rest (*p* < 0.001, *k* = 11). Significant clusters were found with peak coordinates in the pSTS/MTG, left insular cortex and several prefrontal regions with focus on the frontal gyrus (FG) (Table 3; Figure 4A).

**Table 3:** **Adult affective-motivational ROIs and activations in response to Arm touch in all age groups**.

**Adult ROI**	***x,y,z***	**Nr Voxels**	**Adults**		**Adolescents**		**Children**	
			***T***	***p***	***T***	***p***	***T***	***p***
L insula	−37, −8, 12	39	6.13	<0.001	7.46	<0.001^*^	3.37	0.008^*^
R pSTS/MTG	56, −50, 3	34	5.12	<0.001	2.91	0.02^*^	2.51	0.033
R inferior FG	29, 31, 9	97	5.99	<0.001	2.55	0.034^*^	1.88	0.093^†^
R superior FG	11, 49, 27	19	6.05	<0.001	1.97	0.084^†^	1.53	0.160^†^
L medial FG	−7, 28, 30	17	5.17	<0.001	2.09	0.07^†^	1.96	0.081^†^
L superior FG	−10, 22, 51	16	6.11	<0.001	2.09	0.07^†^	1.47	0.177^†^

Talairach x,y,z coordinates and statistics refer to the voxel with the maximum signal change in each region. Abbreviations: region of interest (ROI), right (R), left (L), superior temporal sulcus (STS), medial temporal gyrus (MTG), frontal gyrus (FG).

† Not significant (p > 0.05).

* Significant in whole-brain analysis (Table 1).

The adult pSTS/MTG and left insula ROIs were robustly activated in children and adolescents (Table 3), and the magnitude and temporal dynamics of the brain responses were similar to the adults (Figures 4B,C). With the exception of the right inferior FG, which was significantly activated in adolescents, none of the prefrontal regions were significantly activated in children or adolescents. Nevertheless, the temporal dynamics were similar in all age groups (Figure 4B) and a *post-hoc* ANOVA showed that there was no significant effect of age group on the brain responses to Arm touch in either of the ROIs, including the prefrontal regions (all *ps* > 0.1; Figure 4C). Moreover, there were no significant correlations between age and brain response in any ROI (all *ps* > 0.1; Figure 4D). Finally, there were no significant differences between females and males in adults or children/adolescents (all *ps* > 0.1).

### Whole-brain correlation analysis

**Figure 5:**
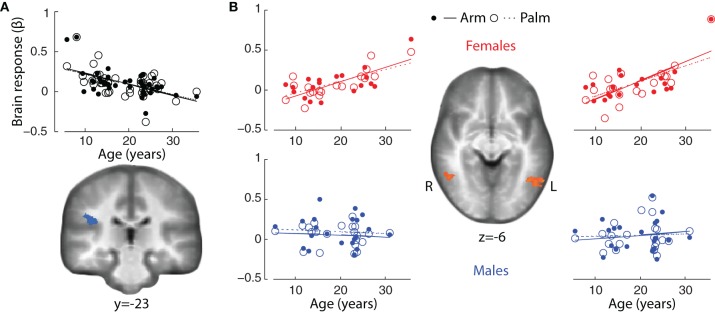
**Correlations between brain responses to tactile stimuli (β values) and age in (A)** ipsilateral SII (*p* < 0.01, *k* = 12) (both genders combined) and **(B)** bilateral pSTS/MTG in females (*p* < 0.01, *k* = 15). The correlation plots show male brain responses extracted from the female regions, and include all males (including those excluded from the statistical analysis).

In both genders combined, there were significant correlations between age and main effect of touch (Arm touch + Palm touch; *p* < 0.01, *k* = 12) in the right (ipsilateral) parietal operculum of SII, likely in the cytoarchitectonic region OP1 (Eickhoff et al., 2006) (negative correlation; Figure 5, Table 4) and the left middle occipital gyrus (positive correlation) (Table 4). Brain responses to stroking of the arm did not reveal any significant correlations beyond the right SII (Table 4).

**Table 4:** **Regions showing a significant correlation between brain responses to touch and age**.

	**Contrast**	**Region**	***x,y,z***	***r***	***p***	**Nr Voxels**
All (*n* = 41)	**Arm + Palm > Rest**	R SII	41, −26, 27	−0.57	0.000	17
		L middle occipital gyrus	−55, −62, −9	0.49	0.001	12
	**Arm > Rest**	R SII	41, −20, 24	−0.51	0.001	13
Females (*n* = 19)	**Arm + Palm > Rest**	R pSTS/MTG	44, −50, −3	0.77	<0.001	26
		L pSTS/MTG	−58, −56, −6	0.74	<0.001	33
		R precuneus	17, −62, 33	0.71	0.001	17
		R cerebellum	17, −62, −21	0.74	<0.001	17
		R cerebellum	14, −44, −39	0.77	<0.001	27
		R cerebellum	14, −44, −39	0.77	<0.001	27
	**Arm > Rest**	L pSTS/MTG	−40, −56, −6	0.73	0.000	19
Males (*n* = 19)	*n.s.*					

Talairach x,y,z coordinates and statistics refer to the voxel with the maximum signal change in each region. Abbreviations: right (R), left (L), secondary somatosensory cortex (SII), posterior superior temporal sulcus (pSTS), medial temporal gyrus (MTG).

The gender-specific analysis revealed strong positive correlations between brain responses in females and age in bilateral pSTS/MTG, the right precuneus and right cerebellum (Arm touch + Palm touch; *p* < 0.01, *k* = 15; Table 4, Figure 5B). The left pSTS/MTG was ventral to, but overlapping with, the adult pSTS/MTG ROI. The correlations were partially driven by a potential outlier (Figure 5B). Nevertheless, excluding this individual still yielded significant correlations in bilateral STS (R pSTS/MTG; *r* = 0.67, *p* = 0.002; L pSTS/MTG; *r* = 0.58, *p* = 0.012). Activity in these regions did not correlate with age in males, on the whole-brain level (Table 4, Figure 5B) or within the female ROIs (all *ps* > 0.1). Moreover, the female correlation coefficients were significantly larger than the male coefficients (all *ps* < 0.05). The Arm only analysis did not reveal any additional regions.

## Discussion

We examined brain responses to gentle brushing of glabrous and hairy skin across development and found a striking continuity in both sensory-discriminative and affective-motivational processing from childhood through adulthood. We also found sexually dimorphic developmental effects in social brain regions, with males lacking the robust age-related increase in pSTS activity observed in females.

The contralateral (left) SII was robustly activated in all age groups, with significant activity on the whole-brain level in all contrasts. Although not comparably robust, children and adolescents significantly activated adult SI and ipsilateral SII ROIs and there were no significant differences between the age groups in these regions. This finding suggests that the sensory-discriminative, somatosensory network is recruited in an adult-like fashion in children. However, we did find an age-dependent linear decrease in a region located to the ipislateral SII area and a non-significant trend toward a positive increase in SI responses with age. Interestingly, Brodoehl et al. (2013) recently demonstrated a similar pattern of decreased SII activation (bilaterally) and increased SI activation in response to tactile stimuli of the fingers in elderly subjects (62–71 years) compared to younger adults (21–28 years). Our results suggest that this aging-related development may represent a life-long trajectory, possibly reflecting continuous dynamics of inhibition and excitation in somatosensory processing.

The prefrontal cortex was activated to some degree in adolescents, and not in children, suggesting a potential developmental effect in these regions. 10-month old, but not younger, toddlers activate the prefrontal cortex more in response to stimulation by soft velvet than to a wooden stimulus (Kida and Shinohara, 2013a), indicating that prefrontal cortex processing of affective touch develop throughout infancy. The prefrontal cortex continues to mature in older children and into adulthood in terms of dynamic synaptic spine development (Petanjek et al., 2011), and it appears likely that affective touch processing in this region is subject to maturation. However, the lack of activations in children did not translate into significant age group differences and there was no linear correlation between age and brain response in prefrontal regions, rendering the results inconclusive.

We found that children, adolescents and, adults alike robustly activate the posterior insular cortex in response to CT-targeted touch, with similar magnitudes, and no gender differences were identified. Selective CT stimulation in patients lacking Aβ fibers activates the posterior, granular insular cortex (Olausson et al., 2002, 2008; Björnsdotter et al., 2009), corresponding to the cytoarchitecturally defined subregions Ig1 and Ig2 (Kurth et al., 2010). The peak adult insular cortex ROI voxel fell just outside the Ig area (Talairach *x, y, z* coordinates: −37, −8, 12), but the ROI overlapped extensively with Ig2 and the centroid (*x, y, z*: −37.57, −10.59, 8.53) was located in this subregion. It is therefore likely that the activations in children and adolescents in this ROI correspond to previously demonstrated Ig2 responses to CT-targeted stimuli in patients (Olausson et al., 2002; Björnsdotter et al., 2009; Morrison et al., 2011a; Liljencrantz et al., 2013) and healthy participants (Morrison et al., 2011b). To the best of our knowledge, this is the first examination of age related effects in insular cortex processing of CT-targeted touch.

All age groups robustly activated a region extending into the right pSTS in response to soft brushing of the arm, and there was a female-specific correlation between age and brain responses in the bilateral pSTS. The pSTS is a key hub in the network of social cognition regions, and is associated with functions such as social attention (Nummenmaa and Calder, 2009) and the visual perception of human motion (Kaiser et al., 2010, 2012). Developmental studies of the STS has shown that, similar to adults, children aged 4.5–15.3 years old activate the pSTS in response to biological motion, suggesting that some key functions of the STS are in place in middle to late childhood (Vander Wyk et al., 2012). However, younger children exhibited a reduced differential response compared to older children, suggesting an ongoing specialization of the pSTS with age. Similarly, research has shown that auditory STS responses increase in selectivity and spatial focus with age in subjects aged 8 years and older (Bonte et al., 2013). Our results confirm that basic mechanism for processing social touch is present in children aged 5 years and older. Moreover, the robust age-related increase in activity observed in females suggests that the STS increases in neural sensitivity throughout maturation. However, this development was not present in males. Our observation is consistent with a recent study showing a higher rate of cortical thinning in females compared to males aged 6–30 years in the right temporal regions, including the STS (Mutlu et al., 2013). Inter-individual variability of STS processing increases with age, however, suggesting that that the function and morphology of the STS is shaped by individual experience during development (Bonte et al., 2013). For example, touch-avoiding individuals may develop attenuated STS responses to affective touch. Consistent with this notion, pSTS responses to affective touch correlate with autistic traits in healthy adults (Voos et al., 2013). Future studies including subjective information, such as behavioral ratings, personality measures, and previous experiences, are necessary to delineate any potential source of inter-individual variability on the observed developmental effect. Nevertheless, this study is the first to reveal putative sex differences in the development of brain processing of affective touch, and contributes an important piece in the puzzle of sex-specific neurodevelopmental disorders: failure to develop social brain regions in males may result in a sex-specific vulnerability to disorders related to social processing, such as autism. Nevertheless, we did not control for personality traits, social behavior or touch preference that may contribute to the observed gender differences. Further study is required to examine the degree to which STS responses may be modulated by such environmental factors.

### Conflict of interest statement

The authors declare that the research was conducted in the absence of any commercial or financial relationships that could be construed as a potential conflict of interest.
